# *The Organon* and Materia Medica Club of the Bay Cities of California

**Published:** 1896-12

**Authors:** W. E. Ledyard

**Affiliations:** B. A., M. B., M. R. C. S., Eng., Secretary


### *THE ORGANON* AND MATERIA MEDICA CLUB OF THE BAY CITIES OF CALIFORNIA.

The regular semi-monthly meeting was held at the office of Dr. J. M. Selfridge, in Oakland, Friday evening, June 19th, 1896.

Members present: Drs. J. M. Selfridge, A. McNeil, W. E. Ledyard, M. T. Wilson, C. M. Selfridge, and G. J. Augur.

The meeting was called to order at 8.10 by the President, Dr. J. M. Selfridge. The minutes of the previous meeting were read by the Secretary, and, after correction by Dr. Selfridge, were approved.

Dr. McNeil brought up the question of admitting new members, stating that he thought it best to form a junior membership, keeping new members on probation for a year, before allowing them to become regular members of the Club, in order to be certain that only good homœopaths would be granted the privileges of regular membership.

Dr. Wilson moved that the President bring the matter up at the next meeting, when more of the members would be present, and it could be presented as an amendment to the Constitution. Seconded by Dr. Ledyard. Carried.

Dr. J. M. Selfridge then read, from *The Organon,* Sections 149 and 150.

DISCUSSION.

Dr. J. M. Selfridge—Hahnemann evidently discovered here that many patients get well without any medicine.

Dr. McNeil—I have not had a case for years that I could positively diagnose as pneumonia. We are often called to see patients with severe colds, we give the indicated remedy and avert the pneumonia.

Dr. J. M. Selfridge—We are usually not called in such cases until there are fever and other serious symptoms, and we then do have a case of pneumonia.

Dr. McNeil—Acute diseases run a certain course, and the patient either gets well or dies. Chronic diseases are different. When a patient, suffering from a chronic disease, gets well, especially a miasmatic disease, I know that the remedy does the work, as these cases are not self-limiting.

Dr. Ledyard—I have a patient in the southern part of the State. I have treated her for a year without seeing her. The quantity of drugs she had taken before that time was appalling. One physician introduced a stick of Nitrate of Silver into the urethra twice a week for six weeks. Now every time she urinates it smarts, burns, etc. Would not a high potency of *Nitrate of Silver* be a good remedy for her?

Dr. C. M. Selfridge—*Natrum-mur.* might act as an antidote to it.

Dr. Ledyard—I give her remedies as they are indicated. She has improved wonderfully since I have been treating her She has falling of the womb, and, as she says, "a falling of the left ovary." Severe pain, which seems to drag clear down to left ovary and womb from the back of the head, even from the vertex. There are great soreness and throbbing all over the body. There is stomach trouble, too.

Dr. McNeil—A magnificent, voluptuous looking woman came to me about three months ago. The cervical glands were swollen almost level. In relating her history, she said that she began to think she had too much adipose tissue, and having read of Phytoline, she took twelve ounces of the fluid extract. She lost flesh a little, but became very depressed mentally. I thought this a good time to use *Phytolacca*, but it had no effect. *Mercurius* covered the case, and I gave her in three months three doses. I saw her several weeks ago, and she was almost well. This shows that *it is not always the high potency of the same drug that will do good*, but the indicated remedy will be the best.

Dr. Ledyard—How long and how high did you give the *Phytolacca?*

Dr. McNeil—I gave it for more than three months in the 200th potency.

Dr. Augur—The potency was not high enough.

Dr. Ledyard—I have a case now of a woman with great weakness in the chest, with severe burning pain extending from the pit of the stomach to the throat. She took some of "Ayer's Cherry Pectoral." After each dose she became dizzy and had to lie down. She attributed many of her symptoms to the use of the Cherry Pectoral; accordingly I potentized it and gave it to her, and she is very much relieved. "Ayer's Cherry Pectoral" is alleged to be composed of Wine of Antimony, Acetate of Morphia, Bloodroot, Ipecac., and Hydrocyanic-acid, the latter being the active principle of Wild Cherry.

Sections 151–153 were read.

### Discussion.

Dr. J. M. Selfridge—Section 153 is a very important part of *The Organon*. We are all very apt to pay too much attention to loss of appetite, restlessness, etc., and lose sight of the more important symptoms. We should, of course, pay attention to the general symptoms as well as the peculiar ones, as they mean a great deal, but we should not prescribe for these to the exclusion of other more important ones. In mentioning restlessness as a general symptom, I do not mean that it should be looked at slightly, for we should know how to distinguish the restlessness of the different drugs.

Dr. McNeil—In studying out a case it is better to leave out the general symptoms.

Dr. Ledyard—I had a bad case. Intense headache, bursting as if top of head would come off when coughing. The next day it changed to the right side of the head, and there was a sensation as if this side of the head would come off when coughing. It was relieved by holding the head firmly between the hands. The patient had right-ear symptoms as well. The head ached all the time, but was made worse by the cough. He had taken Quinine for some time. I gave *Belladonna*, which was the remedy. *China* is also a remedy for headache relieved by hard pressure.

Dr. Augur—Bryonia would have been a good remedy. It has *worse from cough.* Sensation as if the patient would cough the head off.

Sections 154–158 were read.

### Discussion.

Section 155 gives us a very strong reason why we should not use massive doses. *Belladonna* affects the head and also many other organs, as for instance, the uterus. If there be only a headache, the proper dose will cure it; but if we give too large a dose, there will be enough energy in it to bring up symptoms in some of the other organs.

Dr. Augur—In the *Chronic Diseases* Hahnemann says that when you get immediate relief from a remedy the relief is usually of short duration, and you will have to use another remedy to complete the cure, but this last section seems to read differently.

Dr. McNeil—He means in the *Chronic Diseases* that even the allopathic remedy may relieve but does not cure.

Dr. Augur read from the *Chronic Diseases* to explain his statement.

Dr. McNeil—In other words, he looks with suspicion on a cure that is made too quickly. He thinks that if there is a little aggravation, it proves that the right remedy has been given.

Dr. Ledyard—This has been my experience. I have had some chronic cases in which the relief was speedy, but the symptoms came back again.

Dr. Augur.—I have had almost immediate relief without an aggravation, and the symptoms did not come back again.

Dr. McNeil—I had a case in Indiana of an old lady with eczema rubra. It looked like erysipelas of the face. She had been subject to it for some time. There were great *burning and itching, with aggravation from heat.* I gave her *Psorinum*$^{500}$. In a few days she came into my office and I hardly knew her. The entire redness had disappeared. In four weeks a slight redness came back. I gave her one dose of *Psorinum* and she was cured.

Dr. Ledyard—Is not that the opposite of what we know of *Psorinum* regarding heat? The *Psorinum* patient usually wraps up in the hottest kind of weather.

Dr. McNeil—It was the itching that was aggravated by the weather.

Section 159 was read.

### DISCUSSION.

Dr. J. M. Selfridge—In cases of pleuro-pneumonia I have had relief in fifteen minutes without an aggravation. Also in la grippe when I have given *Rhus* for the aching in the back, limbs, etc.

Dr. McNeil—Perceptible aggravations are the exception with the potencies I use.

Dr. Selfridge—If you repeat the dose too often you will get a decided aggravation.

Dr. Augur—A family used to employ me when I was an allopath, but changed their physician when I began to practice Homœopathy. They had a child eight years old with a strong tendency to take cold, and cough which seemed to hang on for a long time. There was a history of consumption in the family. An allopathic physician had treated for some time. At last a friend of mine treated the child but did no good. They then brought the child to me. *Tuberculinum* has *great tendency to take cold.* I gave one dose of Swan's DMM in the afternoon. At nine o'clock the mother called me up saying that the child had been coughing for one whole hour. I ordered a teaspoonful of hot water, as a placebo. The child soon commenced to improve. Two weeks after, from indiscretion in diet, baths, etc., I had to change the treatment, but the child is now getting along splendidly.

Dr. Ledyard—If I repeat *Belladonna* frequently it is apt to give an aggravation. Belladonna acts very quickly, and often *symptoms which arise suddenly* are speedily relieved by it.

Dr. McNeil—I rarely use *Belladonna.* I think Americans do not need it. It is more suitable for Englishmen.

The meeting was then declared adjourned until the first Friday in July, when the meeting would be held at the office of Dr. George H. Martin, 606 Sutter Street, San Francisco, commencing *The Organon* at Section 160.

W. E. Ledyard, B. A., M. B., M. R. C. S., Eng.,
*Secretary.*

Reported by Eleanor F. Martin, M. D.

